# Psychological resilience in the relationship between family function and illness uncertainty among family members of trauma patients in the intensive care unit

**DOI:** 10.1186/s12888-024-05883-0

**Published:** 2024-07-03

**Authors:** Peilin Yu, Wanzhu Zhang, Shijie Li, Xuan Luo, Hao Chen, Jie Mi

**Affiliations:** 1 Department of Critical Care Medicine, The First Affiliated Hospital of Chongqing Medical University, Chongqing, China; 2 The School of Nursing, Chongqing Medical University, Chongqing, China

**Keywords:** Family members, Illness uncertainty, Family function, Psychological resilience, Trauma, ICU

## Abstract

**Background:**

Severe trauma accounts for a main factor inducing mortality for individuals aged < 45 years in China, which requires admission to intensive care unit (ICU) to receive comprehensive treatment. Family members of patients with unanticipated and life-threatening trauma during their ICU stays often experience psychosocial distress due to illness uncertainty. Previous research has shown that family function and psychological resilience are associated with illness uncertainty, respectively. However, little is known about the current situation and interacting mechanism between family function, psychological resilience, and illness uncertainty of family members for ICU trauma patients. Therefore, this study focused on exploring the current situation and relationships between these three factors in family members for ICU trauma patients.

**Methods:**

The convenience sampling approach was adopted in the present cross-sectional survey, which involved 230 family members for ICU trauma patients from 34 hospitals in Chongqing, China. Related data were extracted with self-reporting questionnaires, which included sociodemographic characteristic questionnaire, the Family Adaptability, Partnership, Growth, Affection and Resolve Scale (APGAR), the 10-item Connor-Davidson Resilience Scale (10-CD-RISC) and the Mishel’s Illness Uncertainty Scale for Family Members (MUIS-FM). Pearson correlation analysis was conducted to examine the correlations between various variables. Additionally, a structural equation model was adopted to assess the mediating effect of psychological resilience on family function and illness uncertainty.

**Results:**

According to our results, family members for ICU trauma patients experienced high illness uncertainty with moderate family dysfunction and low psychological resilience. Family function directly affected illness uncertainty and indirectly affected illness uncertainty through psychological resilience in family members of ICU trauma patients.

**Conclusions:**

Family function and psychological resilience are the protective factors for reducing illness uncertainty. Healthcare providers should take effective measures, including family-functioning improvement and resilience-focused interventions, for alleviating illness uncertainty in family members of ICU trauma patients.

## Background

Trauma is the main global public health burden that is associated with high morbidity and mortality rates [1, 2]. In China, there has been a surge in traumatic injuries with the rapid advancement of modern society, resulting in more than 700 thousand deaths each year in the mainland of China [3, 4]. Trauma is becoming the most common factor related to mortality in individuals aged < 45 years in China, which has induced a substantial burden on both the individuals and their families [5, 6]. For patients, trauma can result in immediate death or serious complications, like hemorrhagic shock, systemic inflammatory response, and multiple organ dysfunction syndrome that require rapidly diagnosis and treatment in intensive care unit (ICU) [7–9]. For families, treating trauma patients in the ICU is an unanticipated and devastating event, as their healthy loved ones abruptly enter a state of urgent critical illness with a higher risk of death and disability, so they are often not well prepared to confront with trauma-related challenges, which may thereby induce psychological distress like depression, anxiety, and illness uncertainty [10, 11]. At the same time, illness uncertainty is regarded as a psychological stressor that can aggravate negative distress, like depression, anxiety, or posttraumatic stress disorder [12], which requires attention from healthcare providers.

Illness uncertainty of family members refers to their perception of inability in processing information and determining things of illness-related events [13], and it is a profound psychosocial stressor for family members in the diagnosis, decision-making and prognosis of trauma patients during their ICU stays [14]. Previous research has illustrated that the high illness uncertainty degree among family members may be related to a greater difficulty in coping, a decreased ability in understanding, inability in adapting, dysfunctional problem-solving strategies, higher psychological stress and poorer quality of life [15–18]. It is acknowledged that family members play an irreplaceable role in collaborating with ICU healthcare providers with regard to substitute decision-making, psychosocial support and ongoing care of trauma patients [19], so their illness uncertainty is not only harmful for their health well-beings, but also for the medical outcomes of trauma patients. In the illness uncertainty theory by Mishel, the conceptual framework is provided for explaining uncertainty occurrence and evolution, which consists of 4 main components including (a) antecedents that generate uncertainty, (b) uncertainty appraisal, (c) uncertainty management and (d) illness adaption [20]. According to the antecedents that generate uncertainty, social support is a core component of structure providers for individuals to accurately interpret the illness-related stimuli, thereby reducing the generation of uncertainty [21]. Family is greatly significant for all people in China, and family-derived social support has a crucial role in illness management of individuals [22–24]. Meanwhile, previous research has illustrated that family with normal function can offer great supports to family members, thereby promoting the happiness to buffer adverse physical, social and psychological results [25]. Furthermore, research has reported that family function is negatively associated with illness uncertainty for chronic kidney disease patients [26]. Thus, all of them indicate that family function is a potential factor for reducing illness uncertainty, and we should explore the mechanism of family function and illness uncertainty in family caregivers for ICU trauma patients, so as to provide theoretical foundation for further management.

Based on the illness uncertainty theory by Mishel, Liu Dan pointed out that psychological resilience influenced the responses of patients in uncertainty appraisal and management [27], consistent with previous studies among family members from emergency department and ICU [21, 28]. Psychological resilience refers to the process to adapt well and grow with stress, adversity, and trauma by the American psychological association [29]. Many studies have also illustrated that strong psychological resilience of family caregivers is directly associated with high caregiver preparedness, mild caregiver burden, as well as good mental health and sleep quality [30–33], so it is almost seen as a positive psychological element to resist the negative effects of illness-related stress [34]. Furthermore, there is some research illustrating that psychological resilience can also exert a certain effect on regulating the relationship among acute procedure anxiety, coping styles, post-traumatic growth and illness uncertainty in individuals [35–37], and these are beneficial for shedding novel lights on illness uncertainty management among family members. The condition of patients with severe trauma is characterized as dangerous, complicated, and changeable, and their unexpected hospitalization in the ICU must be a life-threatening crisis for family members. Based on the above findings, we can infer that the strong psychological resilience among family members of ICU trauma patients can adept well in traumatic situations, and psychological resilience can be a mediator for the relation of family function with illness uncertainty.

**Fig. 1:**
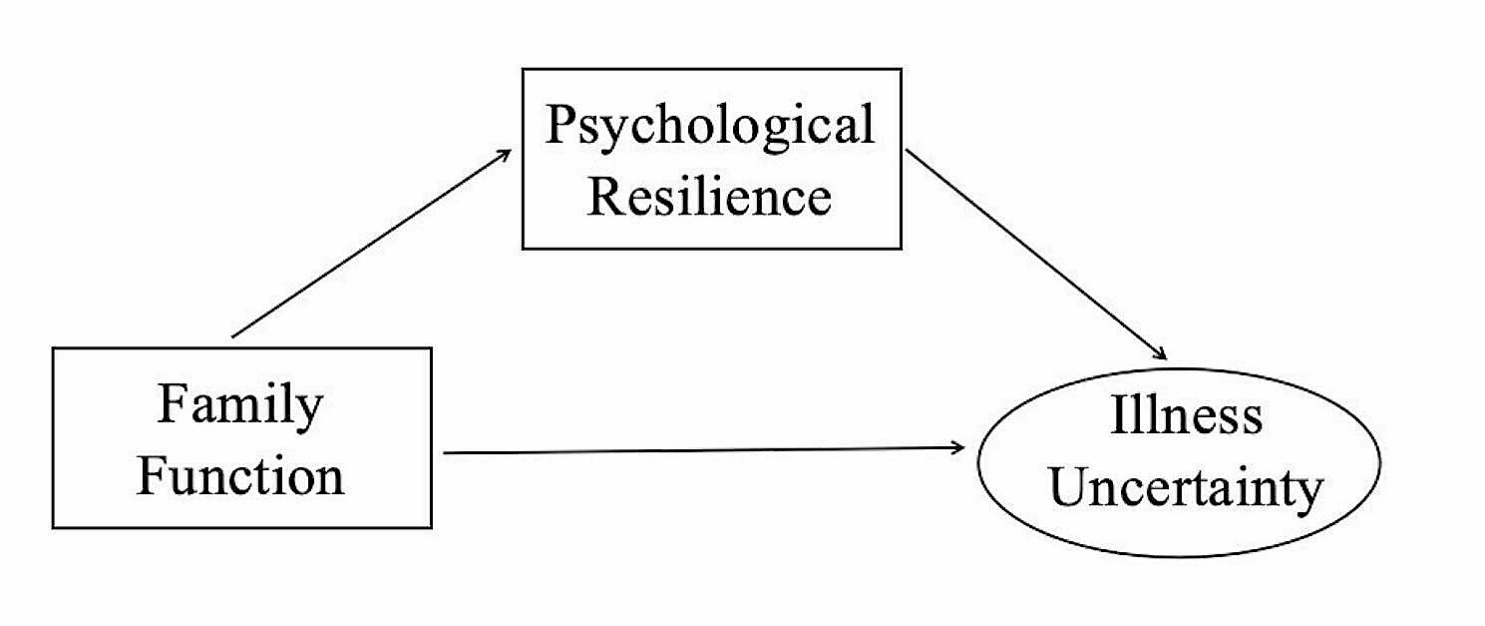
The theoretical model and hypothesis

Although there have been many studies on illness uncertainty in cancer and other chronic disease populations [38–40], little research has focused on illness uncertainty in family members for ICU trauma patients. To our knowledge, although more and more evidence is available in additional fields, relationships among family function, psychological resilience, and illness uncertainty of family members of ICU trauma patients are largely unclear. Therefore, the present cross-sectional survey was performed for investigating the current situation and interacting mechanism between family function, psychological resilience, and illness uncertainty in family members of ICU trauma patients. The present work focused on providing a prevention and intervention conceptual reference framework to assist family members of trauma patients in managing illness uncertainty. Based on exiting research, several hypotheses are put forward: (1) family function is positively associated with psychological resilience, whereas family function is negatively associated with illness uncertainty; (2) psychological resilience shows negative relation with illness uncertainty; and (3) psychological resilience plays a mediating role in the relation of family function with illness uncertainty. Figure 1 displays the theoretical hypothesis model.

## Methods

### Study population

The convenience sampling approach was adopted in the present cross-sectional survey. The study population was family members of ICU trauma patients from 34 public hospitals in Chongqing, China, between September and November, 2022. This study was approved by the Ethics Committee of the First Affiliated Hospital of Chongqing Medical University (Number: 2022 − 203).

### Participants

The inclusion criteria were as follows: (1) family members of ICU trauma patients; (2) aged ≥ 18 years; (3) primarily responsible for decision-making or care-supporting of ICU trauma patients; and (4) adequate reading and communication abilities in Chinese. The exclusion criteria were shown below: (1) participants with cognitive impairments or mental disorders; and (2) formal family caregivers who received payment from patients. Initially, a total of 238 family members completed questionnaires in this work. Nonetheless, 8 non-qualified questionnaires (such as questionnaires that were filled with all same answers) were excluded. Ultimately, 230 family members were included for the analysis.

### Procedure

Prior to the survey, this research project was approved and supported by the head nurse of the ICUs from 34 public hospitals in Chongqing. And 34 professional clinical nurses were designated as the liaisons to collaborate with this research. To ensure the consistency of questionnaire collection, the researcher conducted unified online training and distributed relevant training materials to give detailed explanation on the research contents, criteria of participants, questionnaire completion procedures, item interpretation standards and questionnaire retrieval precautions. After training, random questions were checked to ensure the training quality, and the pre-survey was also conducted to familiarize the procedure and solve preventable problems of data collection. During the survey, the liaisons explained the purpose and method in this questionnaire for obtaining informed consents of family members, and one family member of each trauma patient was selected to complete the paper questionnaires once. After the survey, the questionnaires were collected and examined on the spot to avoid missing any item. Finally, the researcher conducted the statistics and supervision every week, and gave feedback to the liaisons.

### Measurements

The questionnaires included 4 components: (a) sociodemographic data, (b) family function, (c) psychological resilience and (d) illness uncertainty.

### Sociodemographic data questionnaire

The information form covered general sociodemographic data of both family members and patients, such as age, gender, residence, educational, occupation, marital status, monthly income, insurance type, ICU admission round, use of respirator, disability, site of major trauma, and conscious state.

### The family adaptation, partnership, growth, affection and resolve scale (APGAR)

The original Family APGAR scale was compiled by Smilkstein in 1978 [41], and revised into Chinese in 1995 [42], which is used for assessing family function among individuals. Similar to the original family APGAR scale, the Chinese version contains 5 items in 5 dimensions (adaptation, growth, partnership, affection, and resolution). The Likert 3-point scale was utilized, with 0–2 suggesting hardly ever to almost always. The total scores were 0–10 points, with greater scores indicating the greater family function level. The scale scores were divided as 3 categories: severe (0–3) and moderate (4–6) family dysfunction, and good family function (7–10). Cronbach’s α value in this study was 0.798.

### The 10-item connor-davidson resilience scale (10-CD-RISC)

The original 10-CD-RISC scale was compiled via Campbell-Sills in 2007 [43], and revised into Chinese in 2018 [44], which is used to assess psychological resilience of individuals. Similar to the original 10-CD-RISC scale, the Chinese version consists of 10 items with one dimension. The Likert 5-point scale was utilized, with 0–4 suggesting never to almost always. The overall scores were 0–40 points, with greater scores representing the greater psychological resilience level. Cronbach’s α value was 0.887 in this work.

### The mishel’s illness uncertainty for family members scale (MUIS-FM)

The original MUIS-FM scale was compiled by Mishel in 1983 [45], revised into Chinese in 2012 [46], and is currently used to assess illness uncertainty of family members. Different from the original MUIS-FM scale with 30 items, the Chinese version includes 25 items in four dimensions (complexity, ambiguity, unpredictability and information insufficiency). The Likert 5-point scale was utilized, with 1–5 indicating strongly disagree to strongly agree, and items 6, 9, 17, 21, 23, 24 and 25 being the reverse scoring. The overall scores were 25–125 points, with greater scores representing the higher illness uncertainty level. The illness uncertainty of family members was regarded as the high level when the score exceeded 50% of the total score (62.5). In this work, Cronbach’s α value was 0.906.

### Statistical analysis

Statistical analysis was performed using SAS 9.4 and AMOS 24.0 software. Descriptive data were determined by incorporating the percentages, means and standard deviations (SD). ANOVA and t-test were conducted for determining the relations of participants features with family function, psychological resilience, and illness uncertainty. Moreover, we performed Pearson correlation analysis for examining relationships among family function, psychological resilience, and illness uncertainty. The hypothesized model was analyzed using the structural equation model (SEM), whereas model parameters were estimated by the maximal likelihood approach, and Modification Indices were utilized for model adjustment. A good model fit was indicated by χ^2^/df,<3.0, RMSEA < 0.08, GFI ≥ 0.90, IFI ≥ 0.90 and CFI ≥ 0.90. 5000 bootstrap resamples were used to calculate the 95% confidence interval (CI), and *p* < 0.05 stood for statistical significance.

## Results

### Participant data and differences in family function, psychological resilience and illness uncertainty

The age of family members was 20–82 years (mean = 42.12, SD = 11.44). Among them, 202 (87.8%) were married, 124 (53.5%) were unemployed or retired, and 117 (50.9%) had the high school education level. The age of patients was 18–88 years (mean = 54.00, SD = 17.55). Among them, 156 (67.8%) were male, 74 (32.2%) were female, and the majority (69.9%) were unconscious, as shown in Table 1. Further statistical tests revealed that the family members without employment, low monthly incomes and patients with unconscious state were associated with poor family function, low psychological resilience and high illness uncertainty.

**Table 1:** Characteristics of participants and differences in family function, psychological resilience and illness uncertainty (*N* = 230)

Variable	*N*(%)	Label	Family function		Psychological resilience		Illness uncertainty	
			M ± SD	t/F	M ± SD	t/F	M ± SD	t/F
**Age**								
≤ 39	103(44.8)	a	6.97 ± 2.22	0.746	21.1 ± 5.82	1.558	69.45 ± 12.61	0.635
40 ~ 59	117(50.9)	b	6.69 ± 2.39		20.85 ± 5.26		70.27 ± 11.48	
≥ 60	10(4.3)	c	6.2 ± 2.25		17.8 ± 7.87		73.8 ± 12.37	
**Gender**								
male	119(51.7)		6.53 ± 2.31	-1.821	21.71 ± 5.4	2.457^*^	70.33 ± 11.71	0.354
female	111(48.3)		7.08 ± 2.28		19.89 ± 5.79		69.77 ± 12.39	
**Residence**								
city	56(24.4)	a	7.73 ± 2.02^bc^	9.047^**^	22.16 ± 5.65	2.942	69.79 ± 12.2	0.265
county	81(35.2)	b	6.9 ± 2.23^ac^		21 ± 5.69		69.46 ± 11.13	
rural	93(40.4)	c	6.14 ± 2.35^ab^		19.88 ± 5.51		70.74 ± 12.73	
**Education level**								
Elementary school	35(15.2)	a	5.6 ± 2.24^bcd^	4.579^*^	18.09 ± 6.28^bcd^	4.128^*^	73.43 ± 11.79	1.628
Middle school	82(35.7)	b	6.76 ± 2.35^a^		20.99 ± 5.95^a^		68.29 ± 12.2	
High school	66(28.7)	c	7.09 ± 2.2^a^		20.98 ± 5.18^a^		69.79 ± 10.79	
College	47(20.4)	d	7.34 ± 2.17^a^		22.38 ± 4.67^a^		71 ± 13.21	
**Marital status**								
married	202(87.8)		6.75 ± 2.3	-0.761	20.83 ± 5.78	0.222	69.91 ± 11.71	-0.493
Unmarried/divorced/widowed	28(12.2)		7.11 ± 2.41		20.61 ± 4.73		71.11 ± 14.25	
**Occupation**								
employed/business	106(46.1)		7.66 ± 2.1	5.591^**^	22.68 ± 4.57	4.901^**^	67.79 ± 12.29	-2.677^*^
unemployed/retired	124(53.9)		6.06 ± 2.23		19.25 ± 6.02		71.99 ± 11.48	
**Monthly income (RMB)**								
≤ 3000	38(16.5)	a	5.26 ± 2.32^bc^	14.176^**^	16.55 ± 5.55^bc^	18.452^**^	76.24 ± 10.04^bc^	6.433^*^
3001–6000	108(47.0)	b	6.76 ± 2.13^ac^		20.8 ± 5.75^ac^		69.21 ± 11.53^a^	
≥ 6001	84(36.5)	c	7.54 ± 2.19^ab^		22.81 ± 4.43^ab^		68.35 ± 12.68^a^	
**Type of insurance**								
medical insurance	89(38.7)	a	7.58 ± 2^c^	11.195^**^	22.08 ± 5.38^c^	7.217^*^	68.1 ± 11.91^b^	7.075^*^
business insurance	17(7.4)	b	7.29 ± 2.52^c^		23.35 ± 5.65^c^		62.82 ± 13.98^ac^	
no insurance	124(53.9)	c	6.16 ± 2.31^ab^		19.59 ± 5.58^ab^		72.45 ± 11.23^b^	
**Patients’ age**								
≤ 39	53(23.0)	a	6.49 ± 2.32	0.716	20.0 ± 6.34	0.773	68.89 ± 13.07	1.069
40–59	89(38.7)	b	6.81 ± 2.37		21.18 ± 5.62		69.37 ± 12.69	
≥ 60	88(38.3)	c	6.98 ± 2.24		20.96 ± 5.23		71.58 ± 10.42	
**Patients’ gender**								
male	156(67.8)		6.8 ± 2.21	0.054	20.88 ± 5.49	0.210	70.42 ± 12.31	0.659
female	74(32.2)		6.78 ± 2.51		20.72 ± 6.04		69.3 ± 11.43	
**First admission to the ICU**								
yes	209(90.9)		6.77 ± 2.32	-0.524	20.74 ± 5.74	-0.750	70.76 ± 11.87	2.827^*^
no	21(9.1)		7.05 ± 2.22		21.71 ± 4.76		63.1 ± 11.49	
**Use of ventilator**								
yes	149(64.8)		6.45 ± 2.33	-3.143^*^	19.91 ± 5.87	-3.414^*^	72.72 ± 10.66	4.508^**^
no	81(35.2)		7.43 ± 2.13		22.52 ± 4.85		65.16 ± 12.88	
**Disability**								
yes	29(12.6)		5.59 ± 2.1	-3.074^*^	18.62 ± 5.28	-2.271^*^	74 ± 11.94	1.901
no	201(87.4)		6.97 ± 2.29		21.15 ± 5.65		69.49 ± 11.95	
**Major trauma site**								
head and neck	109(47.4)	a	6.45 ± 2.2	1.591	19.76 ± 5.67	2.672^*^	73.61 ± 10.78^bcd^	6.775^**^
thorax and abdomen	63(27.4)	b	7.06 ± 2.36		21.52 ± 5.77		66.84 ± 12.81^a^	
arms and legs	17(7.4)	c	7.24 ± 1.99		21.65 ± 5.43		64.65 ± 13.43^a^	
spine and pelvis	41(17.8)	d	7.12 ± 2.57		22.27 ± 5.15		67.8 ± 10.89^a^	
**Conscious state**								
conscious	70(30.4)		7.66 ± 2.13	3.857^**^	22.9 ± 4.7	4.098^**^	64.14 ± 12.03	-5.21^**^
unconscious	160(69.6)		6.42 ± 2.29		19.93 ± 5.81		72.64 ± 11.1	

Note: T tests and ANOVA analysis, ^*^P < 0.05, ^**^P < 0.01; SNK-q test result, a: compared with layer 1; b: compared with layer 2; c: compared with layer 3; d: compared with layer 4

**Correlation between family function, psychological resilience, and illness uncertainty.**

As reported by family members of ICU trauma patients, the mean(SD) scores of family function, psychological resilience and illness uncertainty were 6.80(2.31), 20.83(5.66) and 70.06(12.02), respectively, as shown in Table 2. According to Pearson correlation analysis, family function showed positive relation to psychological resilience (*r* = 0.626, *P* < 0.01), while negative relation to illness uncertainty (*r* = -0.467, *P* < 0.01). Besides, psychological resilience exhibited negative relation to illness uncertainty (*r* = -0.541, *P* < 0.01).

**Table 2:** Descriptive data and correlation among family function, psychological resilience and illness uncertainty (*N* = 230)

Variable	Family function	Psychological resilience	Illness uncertainty
Family function	1		
Psychological resilience	0.626^**^	1	
Illness uncertainty	-0.467^**^	-0.541^**^	1
M	6.80	20.83	70.06
SD	2.31	5.66	12.02

Note: Pearson correlations, ^*^P < 0.05, ^**^P < 0.01

### Effect of psychological resilience on mediating family function and illness uncertainty

**Fig. 2:**
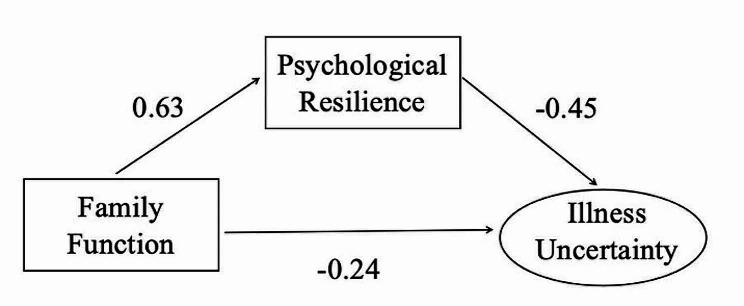
Psychological resilience playing a mediating role in family function and illness uncertainty

As shown by the mediation model results, family function significantly negatively affected illness uncertainty (β = −0.239, SE = 0.076, *P* = 0.002), family function dramatically positively affected psychological resilience (β = 0.626, SE = 0.041, *P* < 0.001), whereas psychological resilience exerted an important negative impact on illness uncertainty (β = −0.445, S.E. = 0.068, *P* < 0.001) (Fig. 2).

Altogether 5,000 bootstrap resamples were conducted for testing model analysis results, which suggested that in “family function → psychological resilience → illness uncertainty” path, psychological resilience achieved an effect value of -0.278 [95%CI (-0.372, -0.196)], besides, 0 was not included in 95%CI, demonstrating the establishment of the mediating effect. Consequently, this hypothesis was verified. Family function has direct influence on illness uncertainty and indirect influence on illness uncertainty via psychological resilience. The total, direct, and mediating effects were − 0.517, -0.239, and − 0.278 separately, with mediating effect occupying 53.77%, as shown in Table 3.

**Table 3:** Bootstrapped point estimates with standard errors and 95% confidence intervals for each indirect effect between family function and illness uncertainty (*N* = 230)

Items	Beta/Effect	SE	*P*	95% CI	
				LLCI	ULCI
**Pathway**					
Family function→Illness uncertainty	-0.239	0.076	0.002	-0.387	-0.091
Family function→psychological resilience	0.626	0.041	< 0.001	0.514	0.701
Psychological resilience→Illness uncertainty	-0.445	0.068	< 0.001	-0.568	-0.306
**Effects**					
Direct effect	-0.239	0.076	0.002	-0.387	-0.091
Indirect effect	-0.278	0.045	< 0.001	-0.372	-0.196
Total effect	-0.517	0.032	< 0.001	-0.623	-0.406

Note: Bootstrap resample = 5000, if the CI does not include zero, the effect is statistically significant at *p* < 0.05

Abbreviations: SE, Standard error; LLCI, Lower Level of Confidence Interval; ULCI, Upper Level of Confidence Interval

## Discussion

This work focused on exploring the current situation and relation among family function, psychological resilience and illness uncertainty of family members for ICU trauma patients. The results showed that the illness uncertainty was high in family members of ICU trauma patients, with moderate family dysfunction and low psychological resilience. Furthermore, family function positively predicted psychological resilience but negatively predicted illness uncertainty, psychological resilience negatively predicted illness uncertainty, and the association between family function and illness uncertainty was partly associated with psychological resilience as hypothesized. Therefore, this work provides theoretical reference for healthcare professionals to take effective measures targeting the improvement of family function and psychological resilience, so as to reduce illness uncertainty in family members of ICU trauma patients, which can finally benefit families’ health well-beings and patients’ medical outcomes.

Illness uncertainty showed an average total score of 70.06 ± 12.02, higher than that of 66.69 ± 12.52 in family caregivers for elderly advanced cancer patients [47]. Such result demonstrated that family members of ICU trauma patients experienced high illness uncertainty, consistent with Hou’s research on families of postoperative lung cancer patients in the ICU [48]. There may be three reasons for this phenomenon. First, most family members of trauma patients are unfamiliar with ICU environment, medical team, procedures and equipment, and the inadequate attention from healthcare providers may increase their feelings of abandonment and helplessness [49], which can lead to high illness uncertainty. Second, the ICU is a specialized medical unit that provides rapidly life-sustaining treatment for critical patients under restricted visitation. Different from the previously restricted policy of allowing families to visit patients at bedsides in the 30-min time limitation once a day, the COVID-19 pandemic during our research has resulted in visiting-cancellation, therefore, family members are not allowed to be present at bedsides, which will affect their ability of coping with illness and thereby aggravate illness uncertainty [50]. Third, most trauma patients are unconscious with complicated and critical conditions due to the interaction of multiple primary injury-causing factors with secondary pathological factors, which require substitute decision-making under an urgent timeframe. However, the inadequate communication with healthcare providers and deficient knowledge about patient treatment may exacerbate their hesitation and doubt, finally giving rise to the high illness uncertainty [51].

Family function showed an average total score of 6.80 ± 2.31, lower than that of 8.80 ± 1.40 in Ying’s research on family caregivers of people with dementia [52], which indicated moderate family dysfunction in family members of ICU trauma patients. There may be two reasons for this phenomenon. First, for patients with severe injuries, their enhanced survival rate has induced high-cost bills on family members, accompanied by a prolonged ICU length of stay and an extended period of rehabilitation at home [53–55]. However, most families are from non-urban districts without employment, their low income and lack of medical insurance can exacerbate the financial strains that are beyond their resources and capacity to cope with illness, as a result, they must be forced to struggle with difficulties alone without sufficient resources. Second, most family members are married middle-aged people who have to shoulder the responsibility of patient ongoing care, children bringing up and family economic sustainability, so the reductions of their social connections and relaxing time can aggravate their emotional strains without pressure release or trouble express [56], eventually leading to the fewer attention and support from families.

The average total score of psychological resilience was 20.83 ± 5.66, which was lower than that of 30.11 ± 0.97 for family caregivers of cancer patients [57]. This phenomenon may be ascribed to two aspects. First, most trauma patients are middle-aged males that are the core mainstay and main labor force in families according to the traditional Chinese culture, so their unanticipated admission to the ICU and the higher possibility of disability due to severe injuries can disrupt the family’s normal life and functions [58], subsequently resulting in biopsychospiritual breakdown for family members towards illness uncertainty and livings in the future. Second, conditions in ICU trauma patients are more unpredictable and changeable than the regular trajectory of cancer, and family members will suffer stressful threats from rapid illness deterioration or failure in life-saving treatment at any time during their ICU stays, which can aggravate their psychological sequelae to impair resilience [59].

As predicted, family function directly affects illness uncertainty and indirectly affects illness uncertainty through psychological resilience. Previous research has shown that maternal illness uncertainty among mothers with very-low-birth-weight preterm neonates in neonatal intensive care unit is negatively correlated with family function, while good family function can promote mutual understanding and cooperation among families to combat the negative challenges related to premature birth, and thereby alleviate maternal illness uncertainty [60]. In addition, Lu’s research on the elderly illustrated that family function was beneficial for their mental health, and they experienced more positive emotions and supports from functional families to build effective psychological resources [61]. Among family members of ICU trauma patients, a good family function can provide strongly emotional, spiritual, economic and material supports to improve their problem-solving abilities, self-efficacy, self-confidence, and positive beliefs [62, 63], which can not only reduce their illness uncertainty but also strengthen their psychological resilience to protect them from traumatic stress. Furthermore, psychological resilience exerts an important effect on mediating illness uncertainty and family function, while psychological resilience is negatively related to illness uncertainty in family members of ICU trauma patients, consistent with previous research on illness uncertainty among stroke patients [37]. Psychological resilience is a protective factor for family caregivers to defend against damages from burden, distress and depressive symptoms, which can empower their abilities to deal with illness-related events [64]. Moreover, previous research has illustrated that resilience acts as a mediating role in improving the quality of life, social support and post-traumatic growth among family caregivers [65–67], which can protect their health well-beings. Similarly, psychological resilience is advantageous for family members of ICU trauma patients, because it can enhance their hope beliefs and problem-focused coping style [68], consequently, they can buffer the effect of illness-related distress to reduce their illness uncertainty.

### Limitations

Several limitations should be noted in this work. First, since this was a cross-sectional survey, we were unable to make definitive conclusions about the causality among the three variables in the SEM. Therefore, future longitudinal or experimental studies are warranted to confirm these findings. Secondly, there might be a self-reporting bias that might have affected these results. Additionally, the data were collected solely in Chongqing, which restricted the generalizability of these findings. Therefore, future large-scale studies from multiple centers across China are needed.

## Conclusions

Illness uncertainty is a major psychosocial stress for family members, which can result in detrimental impacts on medical outcomes of patients and health well-beings of family members. Our research shows that family members of ICU trauma patients show great illness uncertainty with moderate family dysfunction and low psychological resilience. Moreover, our research also shows that family function directly affects illness uncertainty and indirectly affects illness uncertainty through psychological resilience, and the family function and psychological resilience are protective factors for family members of ICU trauma patients to alleviate their illness uncertainty. Therefore, healthcare providers should take effective measures targeting the improvement of family-functioning and resilience-focused interventions for reducing illness uncertainty in family members of ICU trauma patients.

## Data Availability

Data utilized in this work can be obtained from corresponding author upon request.

## Abbreviations

ICU: Intensive Care Unit
APGAR: Family Adaptation, Partnership, Growth, Affection, and Resolve
10-CD-RISC: 10-item Connor-Davidson Resilience Scale
MUIS-FM: Mishel’s Illness Uncertainty Scale for Family Members
SEM: Structural Equation Model
RMSEA: Root Mean Square Error of Approximation
GFI: Goodness-of-Fit Index
IFI: Incremental Fit Index
CFI: Confirmatory Fit Index
